# Repeatability of Neural and Autonomic Responses to Acute Psychosocial Stress

**DOI:** 10.3389/fnins.2020.585509

**Published:** 2020-11-27

**Authors:** Adam M. Goodman, Michael David Diggs, Neha Balachandran, Pranav S. Kakulamarri, Robert A. Oster, Jane B. Allendorfer, Jerzy P. Szaflarski

**Affiliations:** ^1^Department of Neurology, University of Alabama at Birmingham (UAB) Epilepsy Center, University of Alabama at Birmingham, Birmingham, AL, United States; ^2^Department of Medicine, University of Alabama at Birmingham (UAB), Birmingham, AL, United States

**Keywords:** stress, functional magnetic resonance imaging, intraclass correlation, repeatability, psychophysiology

## Abstract

FMRI Montreal Imaging Stress Tasks (MIST) have been shown to activate endocrine and autonomic stress responses that are mediated by a prefrontal cortex (PFC)-hippocampus-amygdala circuit. However, the stability of the neurobehavioral responses over time and the ability to monitor response to clinical interventions has yet to be validated. The objective of this study was to compare the fMRI and physiologic responses to acute psychosocial stress in healthy volunteers during initial and follow-up visits approximately 13 weeks later, simulating a typical duration of clinical intervention. We hypothesized that responses to stress would remain highly conserved across the 2 visits in the absence of an intervention. 15 healthy volunteers completed a variant of control math task (CMT) and stress math task (SMT) conditions based on MIST. Neural responses were modeled using an event-related design with estimates for math performance and auditory feedback for each task condition. For each visit, measures of stress reactivity included differential fMRI and heart rate (SMT-CMT), as well as salivary alpha-amylase before and after scanning sessions. The results revealed that differential fMRI, as well as increased heart rate and salivary alpha-amylase from before and after scanning remained similar between visits. Intraclass correlation coefficient (ICC) values revealed areas of reliable task-dependent BOLD fMRI signal response across visits for peaks of clusters for the main effect of condition (SMT vs CMT) within dorsal anterior cingulate cortex (ACC), insula, and hippocampus regions during math performance and within subgenual ACC, posterior cingulate cortex, dorsolateral PFC regions during auditory feedback. Given that the neurobehavioral response to acute stress remained highly conserved across visits in the absence of an intervention, this study confirms the utility for MIST for assessing longitudinal changes in controlled trials that can identify underlying neurobiological mechanisms involved in mediating the efficacy of stress-reduction interventions.

## Introduction

The endocrine and autonomic responses to acute stress are part of the allostatic process that serves to maintain homeostasis in response to a threat (Ulrich-Lai and Herman, 2009; Karatsoreos and McEwen, 2011). Although this process can be adaptive, dysregulation of the stress response has been implicated in the pathophysiology of a wide range of disorders (McEwen and Gianaros, 2011). Psychosocial stress arising from the threat of social evaluation plays a prominent role in adverse health effects (Dickerson and Kemeny, 2004). Experimental functional magnetic resonance imaging (fMRI) tasks that employ the use of a mild social evaluative stressor have proven a useful tool in the human studies of the neurobehavioral response to stress. In particular, the well-established fMRI Montreal Imaging Stress Task [MIST; (Dedovic et al., 2005)] is used for assessing the neural correlates of psychosocial stress reactivity. Prior studies utilizing MIST have demonstrated that a prefrontal cortex (PFC)-hippocampus-amygdala circuit mediates endocrine and autonomic stress responses (i.e., Pruessner et al., 2008; Dedovic et al., 2009a; Khalili-Mahani et al., 2010; Allendorfer et al., 2014; Wheelock et al., 2016, 2018; Goodman et al., 2019). Despite these contributions to our understanding of the neurobiology of stress, the utility of MIST for assessing longitudinal within-subject changes in reactivity to a common stressor has yet to be validated. Understanding the effects of repeated testing with MIST has important bearing on the prospective utility of this task. In particular, there is potential utility for MIST to assess changes in the neural processing of stressful information arising from clinical interventions, such as cognitive-behavioral therapy (CBT) or mindfulness meditation training (McDermott et al., 2018). Accordingly, demonstrating the validity of MIST to examine the neurobiological benefits of clinical stress-reduction techniques first requires an assessment of the neurobehavioral stress response for potential sensitization (increased) or habituation (decreased) effects that may result from repeated exposure to the task. This new knowledge of the test-retest reliability of MIST will provide valuable insight into the utility of this task for assessing the neurobiological mechanisms underlying stress-reduction techniques.

In MIST, the exposure of participants to varying levels of stressful math tasks allows comparisons between hormonal, autonomic, and neural stress reactivity. However, the effects of repeated assessments of stress-induction create difficulty in disentangling the effect of an intervention. For example, changes in elicited behavior can result simply from repeated exposure to an emotionally evocative stimulus (i.e., non-associative learning). Specifically, increased (sensitization) or decreased (habituation) elicited responses after repeated exposure to a stimulus are mediated by changes in synaptic plasticity (Kandel, 1976). In humans, both sensitization and habituation learning appears to involve changes in activation within PFC-hippocampus-amygdala regions (Breiter et al., 1996; Fischer et al., 2003; Strauss et al., 2005). Accordingly, an assessment of potential sensitization or habituation effects for repeated exposure to MIST is essential to differentiating clinical and learning related changes in neurobehavioral stress response. Examining these potential learning effects across longitudinal MIST assessments in the absence of a clinical intervention will provide novel evidence regarding the utility of MIST for assessing the neurobiological mechanisms underlying stress-reduction techniques. Thus, the objective of the current study was to compare the neural (fMRI) and autonomic (cardiac, alpha-amylase) responses to acute psychosocial stress in healthy volunteers during an initial (V1) and second MRI visit (V2) approximately 13 weeks later, simulating a typical interval before and after a clinical intervention [e.g., 12 weeks of CBT treatment (LaFrance et al., 2014; Espay et al., 2019)]. Although we expected to observe some evidence of non-associative emotional learning, we hypothesized that neural and autonomic responses to stress would remain highly conserved across the 2 visits in the absence of an intervention.

## Materials and Methods

### Participants

Fifteen volunteers (male *n* = 9) with no self-reported history of neurological or psychiatric disorders were recruited from the University of Alabama at Birmingham and completed both study visits. All participants provided written informed consent based on procedures approved by the University of Alabama at Birmingham Institutional Review Board (IRB). The informed consent document provided as much details about the study as possible without revealing the true nature of the study (e.g., “During this scan you will be asked to answer some math questions… About 13 weeks later, you will be asked to return for another visit that includes the questionnaires you completed previously and another MRI.”). Following completion of participation in the study, as per IRB requirement, participants were debriefed with a full explanation of the rationale for the study design and methods used for the study and received $100 for their participation.

### Psychological Measures

Prior to fMRI, all participants completed the 10-item version of the Perceived Stress Scale [PSS-10; (Cohen et al., 1983)] to assess perceived life stress. The PSS-10 is a self-report measure consisting of 10 questions related to stress perception during the month prior to the experimental session scored on a zero (never) to four (very often) Likert scale. PSS-10 scores were computed as a sum, ranging from 0 (little or no stress) to 40 (extreme or high stress), that reflected the degree to which participants found situations or life experiences stressful. Additionally, participants completed the Profile of Mood States [POMS, (McNair, 1992)] to assess affective mood state. The POMS is a self-report measure consisting of 65 questions related to how closely different adjectives described their mood during the week prior to the experimental session. Participants rated each of the 65 adjectives on a zero (Not at All) to four (Extremely) Likert scale. These adjectives provided scores for 6 different mood state subscales: Anger, Confusion, Depression, Fatigue, Anxiety/Tension, and Vigor. POMS scores were calculated by subtracting the Vigor score from the sum of all the other mood scale scores to measure overall mood state (i.e., Total Mood Disturbance; TMD). Possible scores for TMD range between −32 and 200 and reflect the degree to which participants rate their mood disturbance. To assess whether perceived stress or mood states varied between visits, paired samples t-test compared PSS-10 and TMD scores between V1 and V2 assessments.

### Stress Tasks for fMRI

Prior to MRI, participants were familiarized with the MIST task that they later completed during MRI scanning (Balachandran et al., In press). All instructions were scripted to promote uniform administration of the practice and experimental tasks. Participants completed a volume control task during a multi-echo reference scan designed to calibrate audio volume for the remainder of the study. Next, during BOLD Echo-Planar imaging (EPI), participants performed control math (CMT) and stress math tasks (SMT) that were adapted to include pre-recorded evaluative auditory feedback, regardless of performance in the tasks (Allendorfer et al., 2014, 2019; Goodman et al., 2019). Participants selected the correct answer to the math problem via pressing either the “1,” “2,” or “3” button on an MR-compatible button box (Current Designs; Philadelphia, PA, United States). Each of the math task scans contained series of unique math trials, each lasting 5 s in duration. At eight separate fixed points during each of the CMT and SMT scans, unique pre-recorded auditory feedback messages were presented for between 6 and 10 s durations between math trials. For example, participants heard statements such as “You’re doing great, so keep it up” during the CMT and “You will have to do much better in the remaining questions” during the SMT, regardless of their performance in either task. Additionally, auditory recordings of tones (1 s) were presented at eight separate fixed points in which subjects were simultaneously asked to press “1” or “2” on the button box (5 s) to ensure participants were attentive to the task. During the CMT, participants completed 34 different subtraction problems separated by 1.5 s inter-trial intervals. Between the CMT and SMT scans, participants received instructions designed to mildly increase participant’s stress to performing the SMT, compared to the CMT. Specifically, participants were told that “researchers” would be evaluating their performance and they had a variable response window between 1 and 5 s in order for their answer to count. Additionally, participants were given an additional answer choice (3-item multiple choice alternatives) to each math problem and the total number of subtraction problems was increased to 63 trials during the SMT. All other aspects of the SMT were identical to the CMT, including the relative difficulty of subtraction problems. Participant’s accuracy and reaction time during both math and tone trials were recorded and two separate 2 × 2 repeated measures analysis of variance (ANOVA) compared the main effects of Task (CMT vs SMT) and Visit (V1 vs V2), as well as any potential interactions between these factors during math trials. Accuracy and reaction time during math performance and tone events were assessed as a manipulation check to confirm that task performance varied between CMT and SMT as designed.

As in all prior studies, the order of CMT followed by the SMT was identical for all subjects and was not counterbalanced (Allendorfer et al., 2014, 2019; Goodman et al., 2016, 2019; Wheelock et al., 2018; Orem et al., 2019). As demonstrated repeatedly in the stress literature, the acute stress response takes up to 90 min to recover to baseline levels (Kirschbaum et al., 1993; Kudielka et al., 2004; Dedovic et al., 2005; Gaab et al., 2005). If implemented, counterbalancing the order of stress and control scans would be expected to lead to significant variability in brain activity during the control condition simply due to counterbalancing scan order. This design was optimal for detecting individual differences in fMRI signal between SMT and CMT conditions as a function of repeated exposure to the task and scanning.

### Physiological Measures

Heart rate (HR) was recorded with data sampled at 50 Hz from attachment of a photoplethysmograph to the index finger of the left hand. Average beats per minute (BPM) for CMT and SMT conditions for each subject were calculated as two individual mean values based on the entire duration of each task using QRSTool software. One participant was excluded from the heart rate analysis due to equipment failure. Thus, 14 participants were included in heart rate analyses. In order to assess whether cardiac reactivity was greater during the SMT than the CMT, and whether this difference varied as a function of visit, a 2 × 2 repeated measures ANOVA and Bonferroni corrected post-hoc analyses compared the main effects of Task (CMT, SMT) and Visit (V1, V2) on HR, as well as any potential interactions between these factors. An important goal of the current study was to assess potential stress reactivity during both V1 and V2, independently. As in previous work (Allendorfer et al., 2014, 2019; Goodman et al., 2018, 2019; Orem et al., 2019), a preplanned contrasts of SMT and CMT (SMT – CMT) served as an index of increased BPM during stressful compared to control math conditions. In order to assess whether HR was greater during SMT compared to CMT during both visits, a *priori* planned contrasts compared HR for Task (CMT, SMT) at each level of Visit (V1, V2). Given the relatively modest sample size (*n* = 14) for participants that were included in the heart rate analyses, follow-up non-parametric tests (i.e., Wilcoxon signed-rank test) further assessed any significant repeated measures effects identified by the initial parametric 2 × 2 ANOVA and post-hoc tests. To provide a more comprehensive comparison of cardiac responses to acute psychosocial stress across longitudinal assessments, we further evaluated the absence of a meaningful effect of Visit (V1 vs V2), on cardiac stress responses (SMT – CMT) using an equivalence test [i.e., two-one sided test, TOST (Lakens, 2017)]. The implemented TOST procedure was based on the paired-samples *t*-test, but instead tests the null hypothesis that repeated measures are significantly different by assessing whether a result is within an upper and lower 90% confidence interval equivalence bound. First, the obtained effect size (Cohen’s *d*_*z*_) was determined based on the mean difference, SD, n, and correlation between Visit (V1, V2). Then, the estimated distribution of the observed Cohen’s *d*_*z*_ was compared to the bounds of the smallest hypothetical effect size of interest (i.e., Cohen’s *d*_*z*_ = ±0.3). Lastly, we tested for a large hypothetical effect size of interest (i.e., Cohen’s d_*z*_ = ±0.9) to provide a broad estimate of effect sizes that would demonstrate evidence of equivalence, based on the data obtained in our sample.

Cortisol, the hormonal end-result of hypothalamic-pituitary-adrenal cortex (HPA-axis) release, aids in homeostasis and can be used to assess the endocrine response to stress in humans (Jankord and Herman, 2008; Gossett et al., 2018). However, salivary alpha−amylase is closely correlated to HPA-axis activity and has recently emerged as a beneficial alternative due to complications in cortisol measures, including time-lag of effects and anticipatory stress, (Granger et al., 2007; Gossett et al., 2018). Thus, salivary alpha-amylase was assessed at three unique time points throughout each experimental session to evaluate the sympatho-adrenomedullary (SAM) autonomic responses as an additional measure of stress reactivity. At each time point, participants provided 1-ml of saliva via passive drool into plastic tubes. Two samples were collected after consenting during pre-scan assessments at 60 and 30 min prior to entering the MRI environment. One additional sample was collected, immediately at 30 min following completion of MRI scanning. The 3 salivary samples from each participant (−60 min, −30 min, +30 min) were stored on ice until being transferred to a −20 degree Celsius freezer following the experimental session. The natural diurnal pattern in humans is characterized by a pronounced drop in the first hour after waking and gradual increase until the afternoon or evening. Based on this report, we followed recommended guidelines for salivary alpha-amylase assessments to account for potential effects related to the time of day (Nater et al., 2007). Alpha-amylase (U/ml) levels were assessed using standard assay kits in duplicate and averaged at the UAB metabolism core using a standard kit (Salimetrics, LLC) to index SAM responses (Petrakova et al., 2015). One participant did not provide usable saliva and was excluded, resulting in a total of 14 participants that were included in the final alpha-amylase analysis. A 2 × 3 repeated measures ANOVA and Bonferroni-corrected post-hoc analyses assessed the main effects of Time-points (−60 min, −30 min, +30 min) and Visit (V1, V2) on alpha-amylase concentrations, as well as any potential interactions between time point and visit. In order to assess whether alpha-amylase concentrations were greater after MIST compared to before MRI scanning during both visits, a *priori* planned contrasts compared alpha-amylase concentrations for Time-points (−60 min, −30 min, +30 min) at each level of Visit (V1, V2). Given the relatively modest sample size (*n* = 14) for participants that were included in the salivary alpha-amylase analyses, follow-up non-parametric tests (e.g., Wilcoxon signed-rank test) further assessed any significant increases identified by the initial parametric 2 × 3 ANOVA and post-hoc tests. Equivalence of Visit (V1 vs V2) on any significant increases alpha-amylase within both Visits 1 and 2 were also evaluated using the TOST procedure.

### Magnetic Resonance Imaging Acquisition and Analysis

Head-first supine MRI scans were completed on a 3T Siemens Prisma scanner (Siemens Medical Solutions USA Inc., Malvern, PA, United States) at the Civitan International Neuroimaging Laboratory at the University of Alabama at Birmingham. Participants were fitted with an MR-compatible button box (right hand) and viewed a mirror affixed to the head coil that reflected a video monitor (BOLDscreen 32, Cambridge Research Systems ltd., Kent, United Kingdom) in the Siemens scanner. The duration of scanning sessions lasted approximately 60 min. All MRI sessions were scheduled to begin during afternoon hours between 1,300 and 1,700 h.

High resolution T1-weighted anatomical scans were collected in the sagittal plane via magnetization-prepared rapid acquisition with gradient echo (MPRAGE) sequence (TR = 2,400 ms, TE 2.22 ms, TI = 1,000 ms, flip angle = 8°, FOV = 24.0 cm × 25.6 cm × 16.7 cm, matrix = 256 × 256, slice thickness = 0.8 mm)]. The task scans began approximately 45 min from the start of scanning sessions. During task scans, blood-oxygen-level-dependent (BOLD) fMRI signal was measured with a multiband gradient-echo echoplanar pulse sequence (TR = 1,000 ms, TE = 35.8 ms, flip angle = 60°, FOV = 26.0 cm × 26.0 cm × 15.0 cm, matrix = 260 × 260 slice thickness = 2.5 mm, multiband acceleration factor = 6).

Analysis of all MRI data was completed using Analysis of Functional Neuroimaging [AFNI; (Cox, 1996)]. FMRI time-series data were slice-time corrected, corrected for head motion, spatially smoothed with a 4 mm full-width-at-half-maximum Gaussian filter, and co-registered with the structural image (see Supplementary Material for pre-processing scripts). Additional motion correction was performed by censoring images with simultaneous signal change that surpassed 3% of the total number of voxels. Head motion was calculated for each participant visit by averaging the absolute values for displacement (mm) in the superior, left, and posterior directions across all volumes of CMT and SMT scans (i.e., mean absolute head motion) using motion estimates derived during registration of the fMRI time-series (*align_epi_anat.py, 3dvolreg*). Noise occurring outside of the brain was removed using binary masking. Anatomical and functional data were normalized to the MNI 152 ICBM template and resampled to a 1 mm^3^ isotropic resolution. FMRI signal time series from both math tasks were concatenated and then modeled with a gamma variate hemodynamic response function using individual reference waveforms for task events including math trials, audio feedback, and tones for the CMT and SMT (*3dDeconvolve*). The six parameters of participants’ head motion were modeled as regressors of no interest. No other nuisance regression was performed during first-level modeling. Thus, there were 12 total regressors included in first-level modeling, including task relevant and nuisance factors that occurred in the time series. Percent signal change (% signal) was used as an index of the amplitude of the fMRI signal response to task events. Although responses to tone events were included in first-level modeling, these data were not submitted for further analysis in the current study.

Two separate linear mixed-effects analyses (*3dLME*) assessed the neural response to stressful math trials and negative auditory feedback. The first 3dLME analysis identified voxels with a main effect of condition (CMT, SMT), a main effect of visit (V1, V2), or voxels with significant interactions for these variables during math trials. The second *3dLME* analysis identified voxels with a main effect of condition (Positive Feedback, Negative Feedback), a main effect of visit (V1, V2), or voxels with significant interactions for these variables during auditory feedback. A gray matter mask restricted the analysis to the combined regions of interest (ROIs), including the bilateral anterior cingulate, posterior cingulate, insula, dlPFC, dmPFC, vmPFC, vlPFC, amygdala, and hippocampal complex (i.e., hippocampus and parahippocampal gyrus), generated using the standard Harvard-Oxford atlas^1^ (see Supplementary Figure 1 for depiction of the combined ROI mask). ROIs were based on *a priori* hypotheses derived from prior MIST literature (Pruessner et al., 2008; Allendorfer et al., 2014, 2019; Goodman et al., 2016, 2019; Wheelock et al., 2016). A cluster volume extent threshold was determined by the results of a Monte Carlo simulation (*3dClustSim*) in order to reduce risks of family-wise error (FWE) for the combined ROI mask. Smoothness was averaged across subjects based on spherical autocorrelation function parameters (*-acf* option in *3dFWHMx*) derived from residual volumes from the first level analysis (Cox et al., 2017). The results of this simulation yielded a critical cluster extent volume threshold of 88 mm^3^ using a corrected significant threshold *p* < 0.05 and uncorrected voxelwise significance threshold of *p* < 0.001 corresponding to AFNI’s cluster-forming options for nearest neighbor 3 (NN3) with two-sided criteria.

Two separate follow-up analyses were implemented to test the repeatability of task-dependent BOLD fMRI that remained consistent across visits for (1) math performance and (2) audio feedback. First, two separate cluster masks were derived from the surviving clusters identified by *3dLMEs* for the main effect of Condition. To quantify the reliability of the measurements within-subjects, a voxel-wise intraclass correlation (ICC) analysis was performed (Shrout and Fleiss, 1979) using two separate voxel-wise ICC analyses [*3dICC* (Chen et al., 2018)]. ICC values can range between 0 (low consistency) to 1 (high consistency), with ≥0.40 indicating reliable BOLD signal measures across fMRI data acquisition (Cicchetti, 1994; Szaflarski et al., 2011; McDermott et al., 2018). In the current study, two separate *3dICC* analyses with a mixed-model specification [i.e., ICC(3,1)] compared 1st-level coefficients from (1) math performance and (2) auditory feedback events across fixed factors of condition (CMT, SMT) and visit (V1, V2), and the random factor of subjects (*n* = 15). Resulting ICC values were identified for each cluster peak voxel. For descriptive purposes, signal extractions were performed (*3dROIstat*) on representative cluster peaks and mean BOLD signal (% change) across Condition and Visit were plotted to visualize consistency and directional differences in activation for cluster peak regions for any main effects or interactions identified by the two main *3dLME* analyses. Signal extractions were not submitted to additional statistical comparisons because these comparisons were identified as statistically significant by the two main omnibus *3dLME* tests. Accordingly, the purpose of these extractions was to interpret the direction for any significant main effects of Condition, as well as depict potential consistency for the mean estimates of these effects across Visit.

In order to provide further information on the utility of the MIST in assessing treatment mechanisms for future studies, we also calculated estimates of minimum treatment effects that would be needed to overcome the expected neural response variability between visits. More specifically, we estimated variability between visits by first calculating the standard deviation of the differences between visits obtained from results in the current study. The minimum Cohen’s *d* effect size is defined as the expected difference in mean stress response (SMT – CMT; Neg – Pos) between visits (V2-V1) divided by the standard deviation of the difference in means, given our obtained sample size (*n* = 15), 80% power to reject the null, and a two-tailed α = 0.05 threshold. Using the obtained mean difference for any region that met our reliability threshold (i.e., ICC ≥ 0.4), we calculated the minimum mean difference (V2-V1) that is needed to detect a statistically significant effect assuming our obtained sample size, 80% power to reject the null, and a two-tailed α = 0.05 threshold for a paired t-test of the mean difference in stress response between Visits. All power calculations were performed using nQuery Advisor + nTerim (ver. 3.0).

### Data Availability

Unthresholded statistical maps of the results from this manuscript have been made publically available at https://neurovault.org/collections/RPKVOUQF/.

## Results

### Participants

Demographic variables are summarized in Table 1. Years of age were normally distributed (*M* = 32.00, *SD* = 9.03). The durations between V1 and V2 were approximately 13 weeks (*M* = 12.98 weeks, *SD* = 1.34). Two participants chose not to respond to the years of education question. Years of education for the remaining 13 participants were normally distributed (*M* = 16.06, *SD* = 2.03). Additionally, 11 participants identified as “White or European,” while 3 volunteers identified as “Black or African” and 1 identified as other categories (i.e., “American Indian/Alaska Native/Black”).

**TABLE 1:** Demographics, Psychological, Control Math Task, and Stress Math Task, by Visit.

Demographics	Overall	Psychological and task measures	Visit 1 vs Visit 2		
			
			Visit 1	Visit 2	Mean diff
Sample size	*n* = 15	Initial saliva time-point (h:m)	13:51 (1:21)	13:12 (0:59)	0:38
Age	32.00 (9.03)	*Psychological assessments*			
Sex (male)	*n* = 9	PSS-10	14.33 (6.44)	14.00 (7.99)	0.33
Duration between visits (weeks)	12.98 (1.34)	POMS (TMD)	24.60 (31.65)	26.73 (38.20)	−2.13
		***Head motion***			
Years of education	16.46 (2.03)	Mean absolute motion (mm)	0.29 (0.20)	0.28 (0.19)	0.01
		*Control math task*			
Race		Math accuracy (% correct)	97.3 (3.2)	97.3 (3.9)	0.0
White or European	*n* = 11	Response time (ms)	2037.6 (496.1)	1825.0 (358.4)	212.6*
Black or African	*n* = 3	Tone accuracy (% correct)	100.0 (0.0)	100.0 (0.0)	0.0
Other	*n* = 1	Response time (ms)	756.0 (145.8)	780.7 (238.7)	−24.7
		Heart rate (BPM)	60.4 (6.4)	63.6 (8.0)	−3.2
		*Stress math task*			
		Math accuracy (% correct)	61.3 (13.3)	68.3 (13.7)	−7.0*
		Response time (ms)	2878.9 (255.7)	2938.3 (446.2)	59.4
		Tone accuracy (% correct)	98.4 (4.2)	98.4 (4.2)	0.0
		Response time (ms)	704.4 (151.5)	726.3 (209.8)	−21.9
		Heart rate (BPM)	68.9 (16.5)	66.3 (7.9)	2.6

Data for participants reported as mean (SD) except for Sample Size, Sex, and Race which are reported as counts (n). Statistical comparisons were tested using paired samples *t*-tests (*t*) and 2 × 2 repeated measures ANOVA, with Bonferonni corrected *post hocs*. Results of these comparisons, including the mean difference are presented in the adjacent columns to right of the descriptive means and S.D.s for visit 1 vs. visit 2. * indicates any effect or interaction of Visit from the analysis that reached statistical significance (α = 0.05, two-tailed). h, hours; m, minutes; yrs, years; ms, milliseconds; mm, millimeters; PSS, Perceived Stress Scale; POMS, Profile of Mood States; TMD, total mood disturbance.

### Psychological Measures

Participants psychological measures collected at V1 and V2 are summarized in Table 1. PSS-10 scores during were normally distributed during V1 (*M* = 14.33, *SD* = 6.44) and V2 (*M* = 14.00, *SD = 7*.99). Likewise, POMS TMD scores were normally distributed during V1 (*M* = 24.60, *SD* = 31.65) and V2 (*M* = 26.73, *SD* = 38.20). Paired samples *t*-tests revealed that participants PSS-10 *t*(14) = 0.28, *p* = 0.78) and POMS TMD *t*(14) = −0.39, *p* = 0.70) scores did not differ between V1 and V2 assessments.

### Head Motion

Mean absolute head motion (mm) as a function of Visit is reported in Table 1. Paired samples t-tests revealed that participant’s mean absolute head motion did not differ between V1 and V2 assessments [*t*(14) = −0.10, *p* = 0.93].

### Stress Task Measures

Participant’s accuracy and reaction time during math and tone trials as a function of Task and Visit are summarized in Table 1. The 2 × 2 repeated measures ANOVAs revealed a significant interaction between Task (CMT, SMT) and Visit (V1, V2) for math accuracy [*F*(1,14) = 7.20, *p <* 0.05]. Bonferroni-corrected *post hoc* tests revealed this interaction was driven by a difference in V1 and V2 accuracy for SMT (*mean diff* = −7.0, *p <* 0.05), but not CMT (*mean diff* = 0.0, *p* = 1.0). Furthermore, the *post hoc* tests revealed significant differences in CMT and SMT accuracy for V1 (*mean diff* = 36.0, *p <* 0.001) and V2 (*mean diff* = 29.0, *p <* 0.001). The analysis on math response time yielded a significant interaction between Task and Visit [*F*(1,14) = 5.94, *p* < 0.05]. Bonferroni-corrected *post hoc* tests revealed this interaction was driven by a difference in V1 and V2 reaction time for CMT (*mean diff* = 212.58 ms, *p <* 0.05), but not SMT (*mean diff* = −59.44 ms, *p* = 0.51). The analysis failed to yield a significant interaction between Task and Visit for tone response time [*F*(1,14) = 0.00, *p* = 0.95]. Any interactions or main effects involving Visit could not be determined for tone accuracy due to identical mean and standard deviation for Task across Visit (see Table 1). The analysis also revealed significant main effects for Task on math accuracy [*F*(1,14) = 109.35, *p <* 0.001], and math response time [*F*(1,14) = 103.06, *p <* 0.001], but not on tone accuracy [*F*(1,14) = 2.15, *p* = 0.16] or tone response time [*F*(1,40) = 3.71, *p* = 0.08]. Likewise, the analysis revealed a significant main effect for Visit on math accuracy [*F*(1,14) = 7.36, *p <* 0.05], but not on math response time [*F*(1,14) = 1.75, *p* = 0.21], or tone response time [*F*(1,14) = 0.56, *p* = 0.47].

### Physiological Measures

**FIGURE 1:**
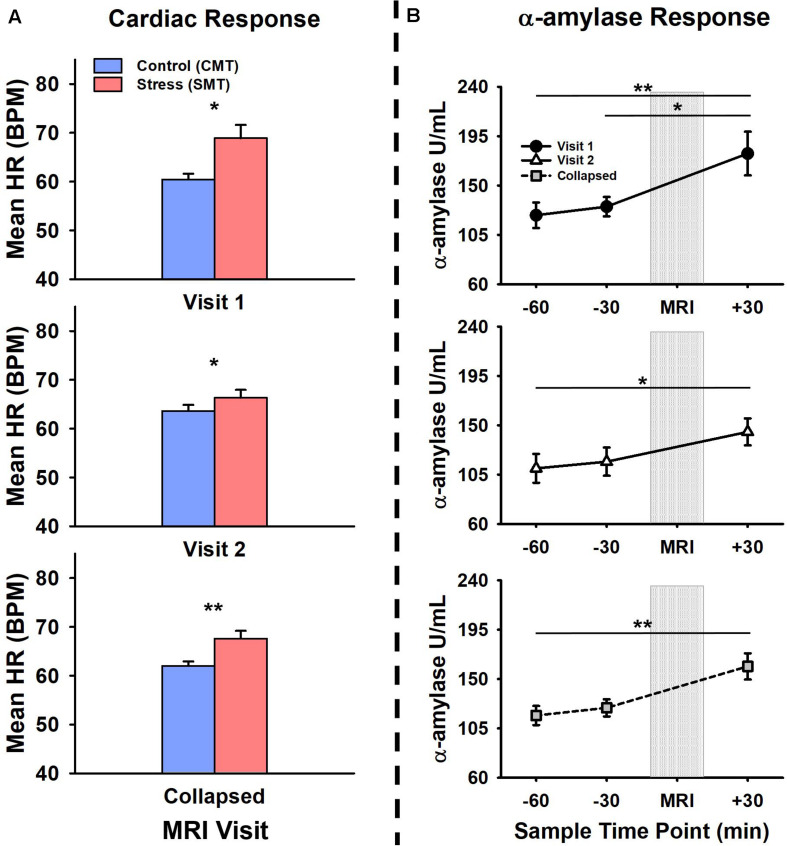
Comparisons of visit 1 (V1), visit 2 (V2), and collapsed (visit 1 and 2 combined) assessments of **(A)** cardiac and **(B)** alpha-amylase (α-amylase; right) stress responses during V1 (top panel), V2 (middle panel), and collapsed across (bottom panel) MRI scanning visits. Heart rate (HR), measured in beats per minute (BPM) was increased during stressful math compared to control math, during V1, V2, and collapsed across visit (a). Salivary α-amylase (U/ml) was increased following stressful math at +30 min post-MRI compared to –60 min and –30 min pre-MRI collapsed across MRI scanning visits. Salivary α-amylase was increased at +30 min compared to –60 min and –30 min during V1, and at +30 min compared to –60 min during V2. Error bars reflect SEM after adjusting for between-subjects variance (Loftus and Masson, 1994). Asterisks indicate significant main effects of condition (SMT vs CMT) on mean HR and time point (–60 min vs +30 min; –30 min vs +30 min) on salivary α-amylase revealed by ANOVA, Bonferonni-corrected *post-hoc*, and planned contrast analyses, *p < 0.05; **p < 0.01.

Figure 1 shows participant’s mean cardiac and salivary alpha-amylase stress reactivity assessments across the MRI visits. Mean BPM results for CMT and SMT across V1 and V2 are summarized in Table 1. The 2 × 2 repeated measures ANOVA that assessed Task (CMT, SMT) and Visit (V1, V2) on HR (BPM) failed to yield a significant interaction of Task and Visit, *F*(1,13) = 2.60, *p* = 0.13, or main effect of Visit, *F*(1,13) = 0.02, *p* = 0.90. This analysis did, however, reveal greater BPM during SMT compared to CMT (*mean diff* = 5.58 BPM), regardless of visit [*F*(1,13) = 10.44, *p <* 0.01]. Planned contrasts revealed greater BPM during SMT compared to CMT during both V1 (*mean diff* = 8.46 BPM, *p* < 0.05) and V2 (*mean diff* = 2.71 BPM, *p* < 0.05). Follow-up Wilcoxon signed-rank tests also revealed greater BPM during SMT compared to CMT during both V1 (*z* = 3.17, *p* < 0.01) and V2 (*z* = 2.97, *p* < 0.01). The TOST procedure indicated that the observed effect size (*dz* = −0.37) was not significantly within the equivalent bounds of dz = ±0.3, (or in raw scores: ±4.48, *t*(13) = −0.25, *p* = 0.60. However, when the anticipated effect of Visit was increased to a much larger effect size of *dz* = ± 0.9, (or in raw scores: ± 13.44), the effect of visit was significantly within the equivalent bounds *t*(13) = 1.88, *p* < 0.05.

The initial salivary sample time-point (−60 min) for all participants (see Table 1) did not differ between visits *t*(13) = 1.38, *p* = 0.19. The 2 × 3 repeated measures ANOVA that assessed Time-points (−60 min, −30 min, +30 min) and Visit (V1, V2), on salivary alpha-amylase (U/mL) failed to yield a significant interaction of Task and Visit, *F*(2,26) = 1.17, *p* = 0.33, or main effect of Visit, *F*(1,13) = 1.37, *p* = 0.26. This analysis did, however, reveal increased salivary alpha-amylase across Time-points (−60 min, −30 min, +30 min), [*F*(2,26) = 9.03, *p <* 0.01]. Bonferroni-corrected *post hoc* tests revealed this significant main effect was driven by increased alpha-amylase measures at +30 min compared to −60 min (*mean diff* = 55.29 U/mL, *p <* 0.01). The remaining Bonferroni-corrected *post-hoc* comparisons failed to reach significance (*mean diffs* < 33.43 U/mL, *ps >* 0.05). Planned contrasts revealed increased alpha-amylase measures at +30 min compared to −60 min (*mean diff* = 70.66 U/mL, *p <* 0.05) and −30 min (*mean diff* = 60.51 U/mL, *p <* 0.01) during V1, and at +30 min compared to −60 min (*mean diff* = 41.91 U/mL, *p <* 0.05) during V2. All remaining planned contrasts failed to reach significance (*mean diffs* < 35.55 U/mL, all *p*s > 0.23). Non-parametric follow-up tests also revealed increased alpha-amylase measures at +30 min compared to −60 min (*z* = 2.73, *p <* 0.01) and −30 min (*z* = 2.61, *p <* 0.05) during V1, and at +30 min compared to −60 min (*z* = 2.10, *p <* 0.05) during V2. Based on the failure to detect a significant interaction between the factors of Task and Visit, but significantly increased alpha-amylase between −60 min and +30 across both Visits 1 and 2, we further evaluated potential time of day effects and equivalence for alpha-amylase stress responses (−60 min > +30 min). There was no effect of time of day on alpha-amylase stress responses (−60 min > +30 min) for Visit 1 (*r* = 0.21, *p* = 0.47) or Visit 2 (*r* = −0.03, *p* = 0.92). The results of the TOST procedure indicated that the observed effect of Visit (*dz* = −0.24) was not significantly within the equivalent bounds of *dz* = ±0.3, (or in raw scores: ±36.5), *t*(13) = 0.24, *p* = 0.41. However, when the anticipated effect of Visit was increased to a much larger effect size of *dz* = ±0.9, (or in raw scores: ±109.56), the effect of visit was significantly within the equivalent bounds *t*(13) = 2.48, *p* < 0.05.

### Magnetic Resonance Imaging Results

**FIGURE 2:**
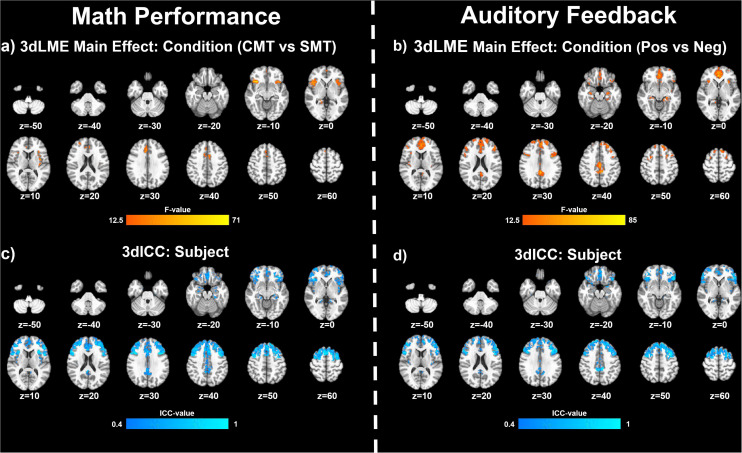
Effects of MIST condition: Clusters (NN3) of significant activation for **(a)** the main effect of Condition (stress math task [SMT] vs control math task [CMT]) and **(b)** for the main effect of Condition (Negative Feedback [Neg] vs Positive Feedback [Pos]) that survived the volume-corrected threshold (uncorrected voxel-wise *p* < 0.001, corrected to α = 0.05). Voxel-wise intraclass correlation (ICC) values (≥0.4) for the subject factor during **(c)** math performance and during **(d)** auditory feedback resulting from 3dLME analyses.

Figure 2 (*top panel*) shows significant clusters identified by the two main *3dLME* analyses that tested for a main effect of Condition (CMT, SMT), a main effect of Visit (V1, V2), or clusters with significant interactions for these factors during math performance events (Figure 2a) and for a main effect of Condition (Positive Feedback, Negative Feedback), a main effect of Visit (V1, V2), or clusters with significant interactions for these factors during auditory feedback events (Figure 2b). Results of the voxel-wise ICC analysis exceeding the pre-determined reliability threshold [ICC ≥ 0.40 (Cicchetti, 1994; Szaflarski et al., 2011; McDermott et al., 2018) are reported in Figure 2 (*bottom panel*) for math performance (Figure 2c) and auditory feedback (Figure 2d). Table 2 reports the regions showing changes in neural response to math performance events during the SMT compared to the CMT that correspond to Figure 2a and the ICC values at peak voxel coordinates that correspond to Figure 2c. Clusters of activation that differed across Condition during math performance trial events were identified with peak voxels located within the dorsal ACC (dACC), PFC, insula, and hippocampus. All clusters for the main effect of Visit and interaction of Task and Visit failed to reach volume-corrected thresholds for math performance trial events. Table 3 reports the regions showing changes in neural response to auditory feedback events during negative feedback compared to positive feedback that correspond to Figure 2b and the ICC values at peak voxel coordinates that correspond to Figure 2d. Clusters of activation that differed across Condition during auditory feedback trial events were identified with peak voxels located within the subgenual ACC, PFC, PCC, hippocampus, and insula regions. All clusters for the main effect of visit and the interaction of Task and Visit failed to reach volume-corrected thresholds. Resultant statistical maps for the main effects and interactions effects within the combined ROI masks without voxelwise or volume-corrected thresholding are presented for Math Performance (Supplementary Figure 2) and Auditory Feedback (Supplementary Figure 3) in the Supplementary Material.

**TABLE 2:** Regions showing effect of condition (CMT vs. SMT) during math performance.

Cluster #	Region	Hemisphere	Vol (mm^3^)	MNI (x,y,z)	*F*-statistic	ICC	V2-V1 diff (obtained)	Min V2-V1 (abs)
**Main effect of condition**								
1	Anterior Insula	R	5335	31, 22, −8	70.92	0.56	−0.09 (0.22)	0.17
2	Dorsal Anterior Cingulate Cortex	R/L	5011	3, 29, 33	53.15	0.63	0.04 (0.37)	0.29
3	Anterior Insula	L	4482	−41, 17, −4	64.64	0.47	−0.1 (0.29)	0.23
4	Ventromedial Prefrontal Cortex	R/L	385	−1, 58, 1	27.35	0.22	–	–
5	Dorsolateral Prefrontal Cortex	R	300	28, 51, 22	33.88	0.66	−0.1 (0.19)	0.15
6	Hippocampus	R	212	19, −40, 1	28.74	0.47	−0.01 (0.28)	0.22

*Cluster #, location, hemisphere, volumes, coordinates from Montreal Neurological Institute (MNI) standard space, *F*-statistic, and Intraclass correlation (ICC) values, difference in BOLD % signal change (SMT-CMT) reported as mean (SD) for V2 - V1, and estimates of a minimum significant treatment effect (absolute value) for the peak voxel of significant clusters. All clusters for the main effect of condition were significant at voxel-wise threshold of *p* < 0.001, corrected to cluster volume threshold of *p* < 0.05 (*3dclustsim* in AFNI).*

**TABLE 3:** Regions showing effect of condition (Positive vs Negative) during audio feedback.

Cluster #	Region	Hemisphere	Vol (mm^3^)	MNI (x,y,z)	*F*-statistic	ICC	V2-V1 diff (obtained)	Min V2-V1 (abs)
**Main effect of condition**								
1	Subgenual Anterior Cingulate Cortex	R/L	32355	−1, 37, −5	77.41	0.45	0.03 (0.71)	0.55
2	Posterior Cingulate Cortex	R/L	9148	−9, −43, 38	84.65	0.72	−0.05 (0.51)	0.39
3	Dorsolateral Prefrontal Cortex	R	3423	44, 11, 28	60.08	0.65	−0.29 (0.46)	0.36
4	Dorsolateral Prefrontal Cortex	L	3128	−37, 8, 31	59.88	0.63	−0.25 (0.53)	0.41
5	Posterior Hippocampus	L	1815	−15, −37, −9	42.99	0.27	–	–
6	Dorsolateral Prefrontal Cortex	R	1516	17, 61, 29	47.62	0.43	−0.13 (0.65)	0.50
7	Dorsolateral Prefrontal Cortex	L	1190	−41, 14, 47	65.76	0.77	−0.21 (0.32)	0.25
8	Dorsolateral Prefrontal Cortex	L	987	−26, 7, 52	43.42	0.67	−0.15 (0.24)	0.18
9	Dorsolateral Prefrontal Cortex	R	941	30, 5, 54	39.57	0.50	−0.14 (0.31)	0.25
10	Ventrolateral Prefrontal Cortex	L	881	−43, 24, −12	36.92	0.68	0.04 (0.74)	0.58
11	Anterior Insula	R	728	34, 23, 3	42.09	0.26	–	–
12	Anterior Insula	L	720	−30, 21, 2	47.94	0.56	−0.06 (0.4)	0.31
13	Dorsomedial Prefrontal Cortex	R	664	13, 36, 54	28.58	0.60	−0.1 (0.26)	0.21
14	Ventrolateral Prefrontal Cortex	R	623	36, 30, −16	29.55	0.38	–	–
15	Anterior Hippocampus	R	574	28, −20, −16	39.58	0.15	–	–
16	Posterior Parahippocampal Gyrus	R	514	21, −32, −16	29.41	0.05	–	–
17	Ventrolateral Prefrontal Cortex	L	224	−34, 61, −12	27.34	0.00	–	–
18	Posterior Insula	R	210	34, −22, 13	33.31	0.49	−0.17 (0.38)	0.29
19	Dorsomedial Prefrontal Cortex	L	186	−7, 13, 54	24.78	0.65	−0.09 (0.3)	0.23
20	Dorsolateral Prefrontal Cortex	L	165	−46, 8, 28	33.05	0.64	−0.4 (0.53)	0.41
21	Posterior Insula	L	145	−34, −25, 18	26.61	0.57	−0.24 (0.43)	0.34
22	Posterior Cingulate Cortex	R	121	8, −47, −2	27.72	0.55	−0.42 (0.64)	0.50
23	Posterior Insula	R	113	39, −9, 9	23.72	0.23	–	–
24	Posterior Insula	L	101	−39, −7, −13	21.89	0.41	−0.16 (0.37)	0.29

*Cluster #, location, hemisphere, volumes, coordinates from Montreal Neurological Institute (MNI) standard space, *F*-statistic, and Intraclass correlation (ICC) values, difference in BOLD % signal change (SMT-CMT) reported as mean (SD) for V2 - V1, and estimates of a minimum significant treatment effect (absolute value) for the peak voxel of significant clusters. All clusters for the main effect of condition were significant at voxel-wise threshold of *p* < 0.001, corrected to cluster volume threshold of *p* < 0.05 (*3dclustsim* in AFNI).*

**FIGURE 3:**
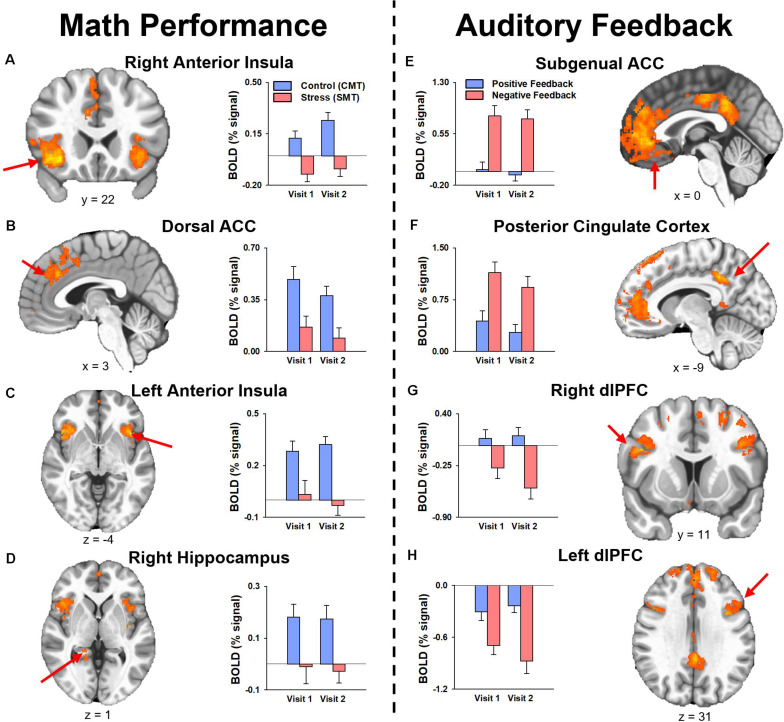
Mean fMRI BOLD percent signal change (% signal) for cluster peaks identified by the analysis for the main effect of Condition with reliable activation across visits (i.e., ICC was ≥0.40, see Tables 2, 3) during **(A–D)** math performance (*left panel*) and **(E–H)** auditory feedback (*right panel*). Mean estimates of effects demonstrated consistency of directional differences in activation for cluster peak regions identified by the two main 3dLME analyses. ACC, anterior cingulate cortex; dACC, dorsal anterior cingulate cortex; dlPFC, dorsolateral prefrontal cortex; PCC, posterior cingulate cortex.

The results of the follow-up analysis revealed areas of reliable task-dependent BOLD signal response across visits within peak voxels of several clusters for the main effect of condition (SMT vs CMT) identified by the initial *3dLMEs*. Figure 3 illustrates the consistency of directional differences for mean BOLD change (% signal) of between Condition (SMT vs CMT; Negative Feedback vs Positive Feedback) in an example subset of cluster peaks in regions with ICC values ≥0.4.

For *n* = 15, 80% power to reject the null, and a two-tailed α = 0.05 threshold for the paired t-test of the mean difference between Visit (H_θ_: V2-V1 = 0), the minimum effect size to detect a significant difference in stress responses (SMT – CMT; Neg – Pos) for all regions is a Cohen’s *d* = 0.778 (i.e., a moderate-to-large effect size). Estimates of the sufficient minimum treatment effects detectable by repeated assessments based on the obtained standard deviations of the difference for regions with ICC values ≥0.4 during Math Performance (Table 2) and Auditory Feedback (Table 3) are reported as the absolute value of the minimum mean difference (V2-V1). The minimum mean difference that is needed to detect a statistically significant difference was always greater than the mean differences observed for these regions.

## Discussion

The primary objective of this study was to compare the neural (fMRI) and autonomic (cardiac, alpha-amylase) responses to acute psychosocial stress in healthy volunteers during two visits separated by approximately 13 weeks simulating a typical clinical intervention duration. Although some evidence of non-associative emotional learning (i.e., sensitization and/or habituation) was predicted, we hypothesized that neural and autonomic responses to stress would remain highly conserved across the two visits in the absence of an intervention. The results indicated that responses to acute psychosocial stress during MIST remained largely consistent between V1 and V2. Further, repeatability analysis demonstrated reliable task-dependent BOLD signal responses across visits. The current study provides evidence that the neural mechanisms underlying autonomic stress responses, as well as these peripheral stress responses themselves, fail to demonstrate evidence of sensitization or habituation as a function of repeated testing with MIST when applied at approximately 13 weeks apart. Given that we observed reliability of task-dependent BOLD signal activation across visits in the absence of an intervention, these finding support the utility of longitudinal assessments of the neurobehavioral response to acute psychosocial stress to assess mechanisms of stress-targeted treatment in randomized controlled trials.

### Stress Task Responses

Accuracy and reaction time comparisons during CMT and SMT conditions are utilized as a manipulation check to validate that task conditions elicited the experimenter-intended psychosocial stress in the task (e.g., Wheelock et al., 2016; Goodman et al., 2019). In the current study, decreased accuracy and increased reaction time (RT) for math trials during the SMT compared to the CMT confirms that task performance varied between MIST conditions as designed. Specifically, decreases in accuracy and increases in reaction time for the SMT compared to CMT did not differ between visits. Alternatively, reaction time was decreased in the CMT and accuracy was increased in the SMT during V2 compared to V1, suggesting that there is a small but significant benefit in task as a function of repeated testing. Given that participants improved in response time during the CMT and providing correct answers during the SMT condition after the initial visit, these results suggests that improvements in reaction time accuracy may reflect repeated testing effects and may not serve as a valid assessment of improvements to stress management. However, it remains unclear whether similar changes in CMT response time or SMT accuracy might occur with a stress-targeted intervention in between assessments. Because we utilized increased task difficulty in the task as part of our experimental manipulation to increase psychosocial stress, there is room for interpretation that differences in accuracy and reaction time during the CMT and SMT tasks include the effects of increased difficulty as well as stress. Difficulty plays an important and interwoven role in this method assessment of the stress response and should be considered in light of future study questions. We propose that any benefits of CMT response time or SMT accuracy that may arise before and after interventions should also be compared to benefits within a control group that does not receive the intervention. Further, it is advised that the emphasis on accuracy and reaction time be placed on validation of the increased difficulty between CMT and SMT tasks, rather than to index any benefit of potential treatment effects.

### Physiological Responses

In addition to math performance measures, participant autonomic responses to MIST are commonly used to validate and index stress reactivity to the task (Allendorfer et al., 2014, 2019; Wheelock et al., 2016; Elbau et al., 2018; Goodman et al., 2018, 2019; Gossett et al., 2018; Orem et al., 2019). In the present study, we sought to assess whether these measures indicated stress reactivity and whether this reactivity varied across repeated MRI visits. Our results indicated that autonomic stress reactivity was both evident and did not differ across the repeated MRI visits. More specifically, the cardiac response to acute psychosocial stress increased during the SMT compared to the CMT, regardless of visit. Likewise, alpha-amylase demonstrated increase in autonomic arousal 30 min post-scanning when compared to the initial sample, 60 min prior to scanning. Although parametric and non-parametric tests demonstrated that these responses did not differ across repeated MRI visits, follow-up equivalence tests failed to demonstrate that these cardiac and alpha-amylase responses to stress were identical across both visits. This failed equivalence result however rests on assumptions of a small-to-modest treatment effect size (i.e., *d_*z*_* = ±0.3) and sample size (i.e., *n* < 15) for these comparisons. We take these findings to suggest that detection of an absent treatment effect via changes in cardiac reactivity and alpha amylase may require relatively larger individual group sample sizes (e.g., *n* > 15) and interventions with moderate-to-strong treatment effect sizes (e.g., *d_*z*_* = ±0.9). Likewise, future studies of treatment effects on the physiological responses during these MRI tasks should be contextualized by comparing changes in stress reactivity between a treatment and control group(s) (see “Limitations” section). Regardless of the initial or repeated visit, cardiac and alpha-amylase reactivity appear to be both reliable and robust indices of autonomic arousal in response to acute psychosocial stress. These results suggest a need for future controlled trials to focus on cardiac and alpha-amylase reactivity to index changes in biobehavioral responses to stress.

### Neural Substrates

Comparisons of fMRI activation between SMT and CMT are often utilized to assess the neural function that underlies biobehavioral responses to acute psychosocial stress during MIST (Pruessner et al., 2008; Dedovic et al., 2009b; Khalili-Mahani et al., 2010; Goodman et al., 2016, 2019; Wheelock et al., 2016; Allendorfer et al., 2019; Orem et al., 2019). Further, comparisons of task-dependent BOLD signal responses between SMT and CMT allow for assessment of unique components of stress reactivity and processing. For example, comparing BOLD signal responses during CMT and SMT conditions related to arithmetic performance may assess inhibitory neural mechanisms related to performance demands, while comparing positive and negative auditory feedback assesses the neural response to extrinsic verbal negative evaluations (Goodman et al., 2019). Thus, the current study aimed to assess whether BOLD signal responses to math performance and auditory feedback differed across repeated MRI visits.

The results indicated that responses to math performance within the dACC, PFC, insula, and hippocampus did not differ across scans and demonstrated a fair to strong (ICC range = 0.47–0.66) degree of repeatability for dACC, dlPFC, insula, and hippocampus activation peaks across visits. Decreased hippocampal activation related to math performance during SMT compared to CMT has been consistently linked to HPA-axis stress reactivity reported in prior MIST literature (Pruessner et al., 2008; Dedovic et al., 2009b; Khalili-Mahani et al., 2010; Goodman et al., 2019). Alternatively, activation of the sympatho-adrenomedullary (SAM) system provokes rapid increases in autonomic activity [e.g., cardiac (Ulrich-Lai and Herman, 2009) and alpha-amylase (Granger et al., 2007)] in response to stress. Activity within PFC and amygdala regions during stressful math vary with cardiac, sweat gland, and self-reported stress (Wheelock et al., 2016; Orem et al., 2019). Further, dACC activity corresponding to bilateral insular activity is also commonly referred to as the *salience network* (Seeley et al., 2007; Uddin, 2015) and has been implicated in emotion regulation studies of reappraisal for negative information (Ochsner et al., 2002; Phan et al., 2005; McRae et al., 2008; Buhle et al., 2014). Thus, the robust and reliable responses to stressful math within the dACC and insular regions observed in the present study implicates this neural network as regions of interest in future studies to assess the neural mechanisms underlying reappraisal-focused CBT interventions.

During auditory feedback, the results indicated that activation within subgenual ACC, PFC, PCC, hippocampus, and insula regions did not differ across scans and demonstrated a fair to strong (ICC range = 0.46–0.71) degree of repeatability for subgenual ACC, PCC, and dlPFC activation peaks across visits. Prior literature that assessed neural function related to auditory feedback during SMT compared to CMT has previously reported differential activation within ACC and PCC regions (Allendorfer et al., 2014; Goodman et al., 2019). Further, ventromedial PFC activity corresponding to PCC activity is also commonly referred to as the *self-referencing network*, and has been implicated in mood disorder studies of self-reflection (Johnson et al., 2002; Whitfield-Gabrieli et al., 2011). Thus, the robust and reliable responses to stressful feedback within subgenual ACC and PCC regions observed in the current study implicates these areas as regions of interest in future studies of the neural mechanisms underlying self-referencing CBT interventions.

### Limitations

Interpretation of the results of the current study should be considered in light of several limitations. First, the study sample was relatively small. However, the chief objective of current study was to assess repeated measures, and the current sample was sufficient to demonstrate reliability estimates in excess of our *a priori* determination for repeatability (ICC ≥ 0.40). **Further, stress responses are known to vary by sex** (Lee et al., 2014). Thus, a significant limitation **of** the current study was that the achieved sample size was not sufficient to assess whether acute stress reactivity was equally repeatable on all measures examined for both sexes. Additionally, the number of subjects with missing SAM reactivity assessments may affect the power to detect significant differences across visits. Although we reported no evidence for the presence of variance in autonomic measures across visits, future studies are encouraged to compare any clinical intervention against a control group for biobehavioral comparisons. Based on the findings from equivalence tests on the biobehavioral measures, relatively low sample-sizes may potentially obscure small treatment effects that differ between such an intervention and control group. Yet, we are unaware of any prior studies utilizing a comparable repeated neuroimaging stress task assessments that would provide a source for anticipated magnitude of such treatment effects. Thus, a lack of estimated treatment effect sizes should be considered a current limitation in our understanding of autonomic response variability across repeated neuroimaging stress task assessments. However, the primary focus of the current report was to validate and assess the repeatability of neural responses to repeated assessment of psychosocial stress reactivity. Related to this point, neural responses revealed no main effects of visit or interactions of condition and visit for math performance or auditory feedback. Therefore, the critical assessment of differential activation of SMT and CMT did not appear to differ across visit. Lastly, the current report did not include a clinical population studied for test-retest reliability without intervention. Our conclusions on the reliability of these neurobehavioral responses are limited to healthy individuals. As a preliminary investigation, however, the current study achieves stated goal of assessing repeated testing effects. Future controlled trial studies can provide further validation of MIST repeatability by showing changes as a function of intervention in clinical populations.

### Conclusion

Given that acute stress responses remained highly conserved across visits, these findings lend support for the utility of MIST to be used to elicit neural and autonomic stress reactivity between repeated, longitudinal assessments. Demonstrating the ability of this task to elicit stress reactivity across longitudinal visits suggests this method, when implemented in a randomized control trial design, may be used to assess changes in neural and autonomic stress responses that underlie the efficacy of therapeutic interventions. In several key brain regions, peak activations of neural responses to stress were reliable between longitudinal assessments approximately 13 weeks apart in the absence of an experimenter intervention. Longitudinal assessments that utilize MIST before and after stress-reduction clinical interventions may provide new knowledge regarding changes in the neural mechanisms of emotion regulation underlying the efficacy of these interventions in clinical populations.

## Data Availability Statement

The datasets presented in this study can be found in online repositories. The names of the repository/repositories and accession number(s) can be found below: https://neurovault.org/collections/RPKVOUQF.

## Ethics Statement

The studies involving human participants were reviewed and approved by the University of Alabama at Birmingham Institutional Review Board (IRB). The patients/participants provided their written informed consent to participate in this study.

## Author Contributions

AMG prepared the manuscript and performed statistical analysis of the data. MDD, JBA, and AMG recruited participants and administered data collection protocols. AMG, MDD, NB, and PSK analyzed and prepared the data for group-level statistics. AMG performed group-level statistics. RAO performed estimate of treatment-effects analysis. AMG, JBA, and JPS designed the study and protocols. All authors contributed to revisions of initial versions of the manuscript.

## Conflict of Interest

The authors declare that the research was conducted in the absence of any commercial or financial relationships that could be construed as a potential conflict of interest.

## Footnotes

1 http://fsl.fmrib.ox.ac.uk/fsl/

## Abbreviations

3dLME: linear mixed effects analysis in AFNI
3dICC: intraclass correlation analysis in AFNI
Abs: absolute value
ACC: anterior cingulate cortex
AFNI: analysis of functional neuroimages
ANOVA: analysis of variance
BOLD: blood oxygen-level dependent
BPM: beats per minute
CBT: cognitive behavioral therapy
CMT: control math task
dACC: dorsal anterior cingulate
dlPFC: dorsolateral prefrontal cortex
dmPFC: dorsomedial prefrontal cortex
fMRI: functional magnetic resonance imaging
HPA: hypothalamic-pituitary-adrenal
HR: heart rate
HC: hippocampal complex (hippocampus and parahippocampal gyrus)
ICC: intraclass correlation coefficient
MIST: montreal imaging stress tasks
mm: millimeters
MNI: montreal neurological institute
MTL: medial temporal lobe
Neg: negative feedback
PCC: posterior cingulate cortex
PFC: prefrontal cortex
POMS: profile of mood states
Pos: positive feedback
PSS: perceived stress scale
RT: reaction time
SAM: sympatho-adrenomedullary
SMT: stress math task
TMD: Total Mood Disturbance
TOST: Two-one sided tests
V1: Visit 1
V2: Visit 2
vlPFC: ventrolateral Prefrontal Cortex
vmPFC: ventromedial Prefrontal Cortex.
