# Supplementary figures and images for: Repeatability of Neural and Autonomic Responses to Acute Psychosocial Stress
Source: Front Neurosci. 2020 Nov 27;14:585509. doi: 10.3389/fnins.2020.585509 (PMC7732671; doi:10.3389/fnins.2020.585509)

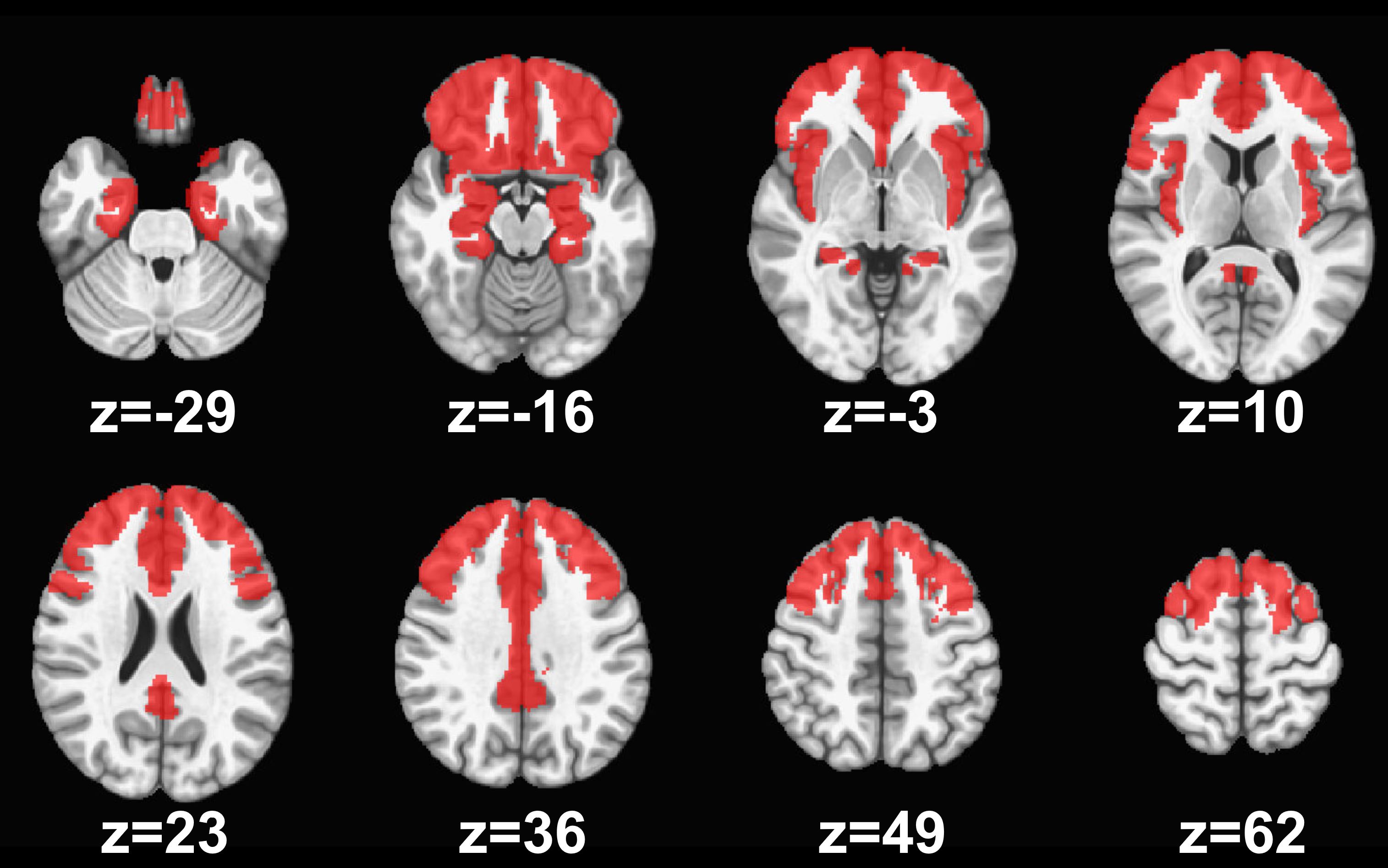

Supplement: Supplementary file 1 [file Image_1.JPEG]

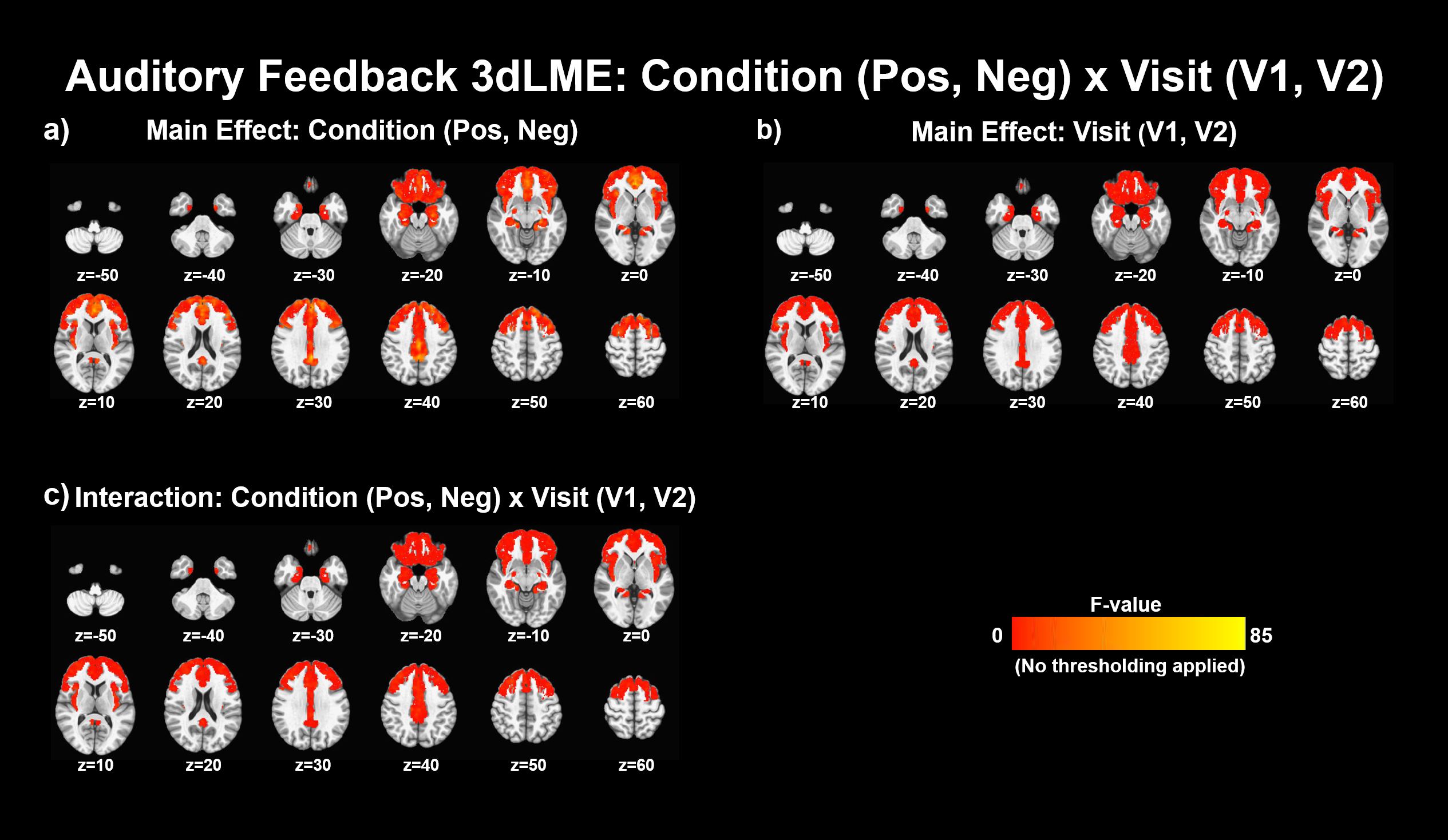

Supplement: Supplementary file 2 [file Image_2.JPEG]

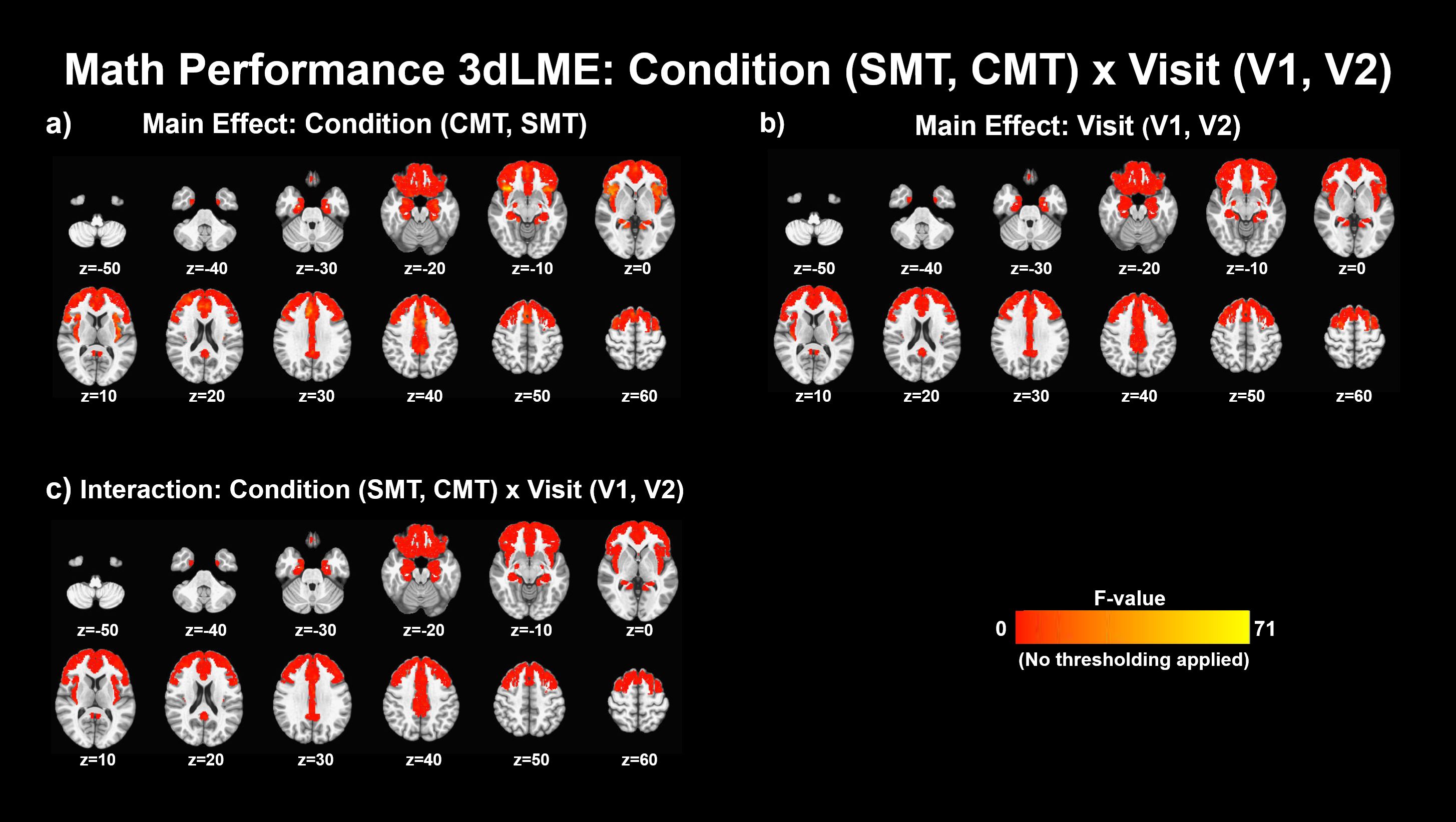

Supplement: Supplementary file 3 [file Image_3.JPEG]
