# Supplementary material for: Repeatability of Neural and Autonomic Responses to Acute Psychosocial Stress
Source: Front Neurosci. 2020 Nov 27;14:585509. doi: 10.3389/fnins.2020.585509 (PMC7732671; doi:10.3389/fnins.2020.585509)
Supplement: Supplementary file 4 [file Table_1.DOCX]

**Supplementary Materials**

**S1. Supplementary Methods**

**S1.1. Preprocessing script**.

!/bin/bash

## Preprocessing stream after converting to .nii

# Creates fMRI data that's aligned to anat, motion-corrected, then

# Creates difle data for inputting as regressors in GLM and

# censoring timepoints with excessive motion (censors TRs w/ >3% motion)

# Set basedir, sub ID, visit number

basedir=C:/basedir

cd ${basedir}/MRI_processed_data

for sub in SUB_ID

do

for vis in VIS_ID

do

cd ${basedir}/MRI_processed_data/${sub}_V${vis}

# Removes skull from Anat \

3dSkullStrip -input ${sub}_V${vis}_anat.nii -prefix ${sub}_V${vis}_anat_ns.nii

#Alignment of Math1 epi and produces motion file

align_epi_anat.py -epi ${sub}_V${vis}_math1.nii \

-anat ${sub}_V${vis}_anat_ns.nii \

-anat_has_skull no \

-epi2anat -epi_base 0 -suffix _aligned \

-volreg_method 3dvolreg -volreg_opts '-Fourier' -dfile ${sub}_V${vis}_math1_motion.difle \

-Allineate_opts '-warp shift_rotate' \

-tshift on \

-deoblique off \

-save_vr

3dToutcount -automask -fraction -polort 3 -legendre \

${sub}_V${vis}_math1_aligned+orig >> outcount.${sub}_V${vis}_math1.1D

#Alignment of Math2 epi and produces motion file

align_epi_anat.py -epi ${sub}_V${vis}_math2.nii \

-anat ${sub}_V${vis}_anat_ns.nii \

-anat_has_skull no \

-epi2anat -epi_base 0 -suffix _aligned \

-volreg_method 3dvolreg -volreg_opts '-Fourier' -dfile ${sub}_V${vis}_math2_motion.difle \

-Allineate_opts '-warp shift_rotate' \

-tshift on \

-deoblique off \

-save_vr

3dToutcount -automask -fraction -polort 3 -legendre \

${sub}_V${vis}_math2_aligned+orig >> outcount.${sub}_V${vis}_math2.1D

#threshold outlier criteria (>3% voxels outliers) & save motion censoring files as (0 or 1) for each TR.

1deval -a outcount.${sub}_V${vis}_math1.1D -expr '1-step(a-0.03)' > 3%${sub}_V${vis}_math1.txt

1deval -a outcount.${sub}_V${vis}_math2.1D -expr '1-step(a-0.03)' > 3%${sub}_V${vis}_math2.txt
